# Association of English language proficiency and breast cancer oncologic outcomes

**DOI:** 10.1093/jncics/pkag079

**Published:** 2026-08-17

**Authors:** Revathi Ravella, Lillian A Boe, Anushri Mahabir, Boris A Mueller, Emma N Hahesy, Jeehee Isabelle Choi, Anyi Li, Simon N Powell, Atif J Khan, Lior Z Braunstein

**Affiliations:** Department of Radiation Oncology, Memorial Sloan Kettering Cancer Center, New York, NY, United States; Department of Epidemiology and Biostatistics, Memorial Sloan Kettering Cancer Center, New York, NY, United States; Department of Neuroscience, Princeton University, Princeton, NJ, United States; Department of Radiation Oncology, Memorial Sloan Kettering Cancer Center, New York, NY, United States; Department of Radiation Oncology, Memorial Sloan Kettering Cancer Center, New York, NY, United States; Department of Radiation Oncology, Memorial Sloan Kettering Cancer Center, New York, NY, United States; Department of Radiation Oncology, Memorial Sloan Kettering Cancer Center, New York, NY, United States; Department of Radiation Oncology, Memorial Sloan Kettering Cancer Center, New York, NY, United States; Department of Radiation Oncology, Memorial Sloan Kettering Cancer Center, New York, NY, United States; Department of Radiation Oncology, Memorial Sloan Kettering Cancer Center, New York, NY, United States

## Abstract

Few studies have compared oncologic outcomes directly between English-speaking (ES) and non-English-speaking (NES) patients. We performed a matched cohort study of stage I-III breast cancer patients with ES patients propensity matched 3:1 to NES patients based on age, tumor stage, nodal involvement, and receipt of adjuvant therapy. Patients were categorized as ES or NES according to preferred language documented in the electronic medical record. Primary outcomes were locoregional recurrence (LRR), disease-free survival (DFS), and overall survival (OS). Among 2768 patients (2076 ES; 692 NES), median age at diagnosis was 62 years (range = 25-94). Ten-year LRR was 9.5% vs 13% (*P *= .032), 10-year DFS was 71% vs 63% (*P* = .008), and 10-year OS was 79% vs 74% (*P *= .12) for ES and NES patients, respectively. These findings suggest that NES patients may have inferior breast cancer outcomes, even in highly resourced academic environments. These findings highlight the need for targeted interventions beyond language assistance.

## Introduction

Language proficiency is increasingly recognized as an important determinant of health. Studies have shown that patients with limited English proficiency (LEP) face systemic barriers across the cancer care continuum, including lower breast cancer screening rates,^1^ delays in diagnosis,^2^ presentation with later-stage disease,^3^^,^^4^ and reduced access to timely treatment.^5^^,^^6^ These barriers likely reflect broader structural inequities—including socioeconomic constraints, immigration-related challenges, and reduced health literacy—that extend well beyond language itself and may cumulatively affect oncologic outcomes. However, no studies have directly compared oncologic outcomes among English-speaking (ES) vs non-English-speaking (NES) patients with breast cancer.^7^^,^^8^

## Methods

We conducted a propensity matched cohort study of patients with stage I-III breast cancer treated with definitive intent at Memorial Sloan Kettering Cancer Center from 2008 to 2019. The study was conducted with approval of the Memorial Sloan Kettering Institutional Review Board (IRB #16-114). Patients were categorized as ES or NES according to preferred language as documented in the electronic medical record at time of registration with reaffirmation throughout their care. Institutional language-access services, including professional interpreter services, were available to patients throughout the study period.

Clinicopathologic, treatment, and demographic data were collected from the medical record. ES patients were matched in a 3:1 ratio to NES patients based on age, tumor stage at diagnosis, nodal involvement, and adjuvant treatment using nearest neighbor propensity score matching without replacement. Absolute standardized mean differences (ASMD) ranged from 0.0005 to 0.0250, indicating the criteria of balance (ASMD < 0.10) was satisfied for all matching variables.

The primary oncologic outcomes of interest were locoregional recurrence (LRR), disease-free survival (DFS), and overall survival (OS). LRR and distant recurrence events were identified in the medical record and confirmed pathologically. Survival was defined by a pipeline cross-referencing the Social Security Death Index, obituary records, and targeted follow-up of patients lost to routine contact. LRR was defined as recurrence in the ipsilateral breast/chest wall or regional lymph nodes. DFS was defined as time from diagnosis to any recurrence (local, regional, or distant) or death. OS was defined as the time from diagnosis to death from any cause. Patients alive at last follow-up were censored at that time.

Variables in the propensity-score matched cohort were compared by using random-intercept logistic regression, accounting for the matched nature of the data. Survival outcomes were estimated using the Kaplan-Meier method and compared by using the log-rank test. Cumulative incidence of LRR was estimated by using competing risks analysis, with death and other recurrence as competing events, and compared by using Gray’s test. After propensity score matching, Cox proportional hazards models were used to estimate hazard ratios for DFS and OS, stratified by the matched pairs. Fine-Gray subdistribution hazards models accounting for clustering within matched pairs were used to estimate hazard ratios for LRR with death as a competing risk. As a sensitivity analysis to assess the potential confounding effect of socioeconomic variables, multivariable models were performed adjusted for race, ethnicity, marital status, religion, and insurance. A *P* value < .05 was considered statistically significant. All statistical analyses were conducted using R version 4.5.0.

## Results

### Patient and treatment characteristics

A total of 2768 patients with nonmetastatic breast cancer were included in the final study cohort, comprising 2076 ES patients and 692 matched NES patients (3:1 ratio). Patient and tumor characteristics are presented in Table 1. The median age at diagnosis was 61.5 years (range = 25.4-93.5). Baseline demographic and tumor characteristics were well balanced between the groups following matching. Rates of breast-conserving surgery, mastectomy, radiation therapy, and chemotherapy use were similar between groups. The most common languages spoken among the NES group were Spanish (*n* = 206, 30%), Russian (*n* = 147, 21%), Arabic (*n* = 61, 8.8%), Mandarin (58, 8.4%), and Cantonese (*n* = 38, 5.5%). Median follow-up time was 84.4 (range = 0.5-360.7) months.

**Table 1.** Patient, tumor, and treatment characteristics in propensity-matched cohort.

Characteristic	Overall *N* = 2768^a^	English *n* = 2076^a^	Non-English *n* = 692^a^	*P* ^b^
Age at diagnosis	61.5 (24.7, 93.5)	61.5 (24.7, 93.5)	61.5 (25.4, 88.9)	.8
Race^c^				<.001
Asian	251 (9.1%)	110 (5.3%)	141 (20%)	
Black	234 (8.5%)	213 (10%)	21 (3.0%)	
Other	84 (3.0%)	26 (1.3%)	58 (8.4%)	
Unknown/refused	203 (7.3%)	97 (4.7%)	106 (15%)	
White	1996 (72%)	1630 (79%)	366 (53%)	
Ethnicity				<.001
Hispanic	280 (10%)	106 (5.1%)	174 (25%)	
Not Hispanic Latino	2448 (88%)	1938 (93%)	510 (74%)	
Unknown	40 (1.4%)	32 (1.5%)	8 (1.2%)	
Marital status				.067
Divorced/separated	278 (10%)	219 (11%)	59 (8.5%)	
Married/partner	1656 (60%)	1254 (60%)	402 (58%)	
Unknown/unspecified	7 (0.3%)	6 (0.3%)	1 (0.1%)	
Unmarried/single	430 (16%)	320 (15%)	110 (16%)	
Widowed	397 (14%)	277 (13%)	120 (17%)	
Religion				
Buddhist	24 (0.9%)	9 (0.4%)	15 (2.2%)	
Christian	1581 (57%)	1244 (60%)	337 (49%)	
Hindu	31 (1.1%)	26 (1.3%)	5 (0.7%)	
Jewish	360 (13%)	277 (13%)	83 (12%)	
Muslim	84 (3.0%)	15 (0.7%)	69 (10.0%)	
None	542 (20%)	402 (19%)	140 (20%)	
Other	27 (1.0%)	21 (1.0%)	6 (0.9%)	
Unknown	119 (4.3%)	82 (3.9%)	37 (5.3%)	
Insurance				
Commercial	643 (23%)	532 (26%)	111 (16%)	
Medicaid	167 (6.0%)	66 (3.2%)	101 (15%)	
Medicare/medicare advantage	1245 (45%)	991 (48%)	254 (37%)	
Other/unknown/self-pay	713 (26%)	487 (23%)	226 (33%)	
Median income by zip code, $	106 741.0 (21 278.0, 250 001.0)	114 845.0 (25 786.0, 250 001.0)	79 136.0 (21 278.0, 250 001.0)	
Unknown	91	20	71	
Breast surgery				>.9
Breast conservation	1764 (64%)	1322 (64%)	442 (64%)	
Mastectomy	1004 (36%)	754 (36%)	250 (36%)	
Axillary surgery				.092
ALN	759 (27%)	552 (27%)	207 (30%)	
SLN	2009 (73%)	1524 (73%)	485 (70%)	
Positive nodes removed	0.0 (0.0, 38.0)	0.0 (0.0, 38.0)	0.0 (0.0, 35.0)	.7
Overall grade				.4
I	274 (9.9%)	216 (10%)	58 (8.4%)	
II	1189 (43%)	892 (43%)	297 (43%)	
III	1047 (38%)	772 (37%)	275 (40%)	
Unknown	258 (9.3%)	196 (9.4%)	62 (9.0%)	
Pathological size (cm)	1.5 (0.1, 19.0)	1.5 (0.1, 14.8)	1.6 (0.1, 19.0)	.053
Pathological size missing	200 (7.2%)	149 (7.2%)	51 (7.4%)	.9
T stage				>.9
T0	99 (3.6%)	74 (3.6%)	25 (3.6%)	
T1	1701 (61%)	1281 (62%)	420 (61%)	
T2	780 (28%)	580 (28%)	200 (29%)	
T3	85 (3.1%)	64 (3.1%)	21 (3.0%)	
T4	10 (0.4%)	8 (0.4%)	2 (0.3%)	
Tis	81 (2.9%)	61 (2.9%)	20 (2.9%)	
Tx	12 (0.4%)	8 (0.4%)	4 (0.6%)	
Lymphovascular invasion				.023
No	1650 (60%)	1267 (61%)	383 (55%)	
Unknown	176 (6.4%)	123 (5.9%)	53 (7.7%)	
Yes	942 (34%)	686 (33%)	256 (37%)	
Multifocal				.2
No	1964 (71%)	1465 (71%)	499 (72%)	
Unknown	1 (<0.1%)	0 (0%)	1 (0.1%)	
Yes	803 (29%)	611 (29%)	192 (28%)	
ER status				.9
Negative	509 (18%)	386 (19%)	123 (18%)	
Positive	2220 (80%)	1661 (80%)	559 (81%)	
Unknown/not done	39 (1.4%)	29 (1.4%)	10 (1.4%)	
PR status				>.9
Negative	763 (28%)	571 (28%)	192 (28%)	
Positive	1930 (70%)	1448 (70%)	482 (70%)	
Unknown/not done	75 (2.7%)	57 (2.7%)	18 (2.6%)	
HER2 status				.5
Equivocal	4 (0.1%)	4 (0.2%)	0 (0%)	
Negative	2243 (81%)	1691 (81%)	552 (80%)	
Not done/unknown	86 (3.1%)	65 (3.1%)	21 (3.0%)	
Positive	435 (16%)	316 (15%)	119 (17%)	
Chemotherapy	1442 (52%)	1086 (52%)	356 (51%)	.7
Hormonal therapy	1982 (72%)	1481 (71%)	501 (72%)	.6
Radiotherapy	1881 (68%)	1406 (68%)	475 (69%)	.7
Local recurrence	172 (6.2%)	118 (5.7%)	54 (7.8%)	
Distant recurrence	331 (12%)	239 (12%)	92 (13%)	
Regional recurrence	70 (2.5%)	47 (2.3%)	23 (3.3%)	
LRR	213 (7.7%)	149 (7.2%)	64 (9.2%)	
Death	421 (15%)	311 (15%)	110 (16%)	
Time to death or last follow-up (months)	84.4 (0.5, 360.7)	85.4 (0.5, 360.7)	77.1 (1.5, 357.8)	<.001
Time to recurrence of any type, death, or last follow-up (months)	78.0 (0.1, 350.5)	80.8 (0.1, 350.5)	70.7 (0.2, 318.3)	<.001

Abbreviations: ALN = axillary lymph node dissection; ER = estrogen receptor; LRR = locoregional recurrences; PR = progesterone receptor; SLN = sentinel lymph node biopsy.

a Median (min, max); *n* (%).

b Random intercept logistic regression.

c Race categories reflect structured electronic medical record entries at the time of registration. “Other” indicates a self-reported race not otherwise listed. “Unknown” indicates data that were unavailable or not recorded.

### Oncologic outcomes

**Figure 1.**
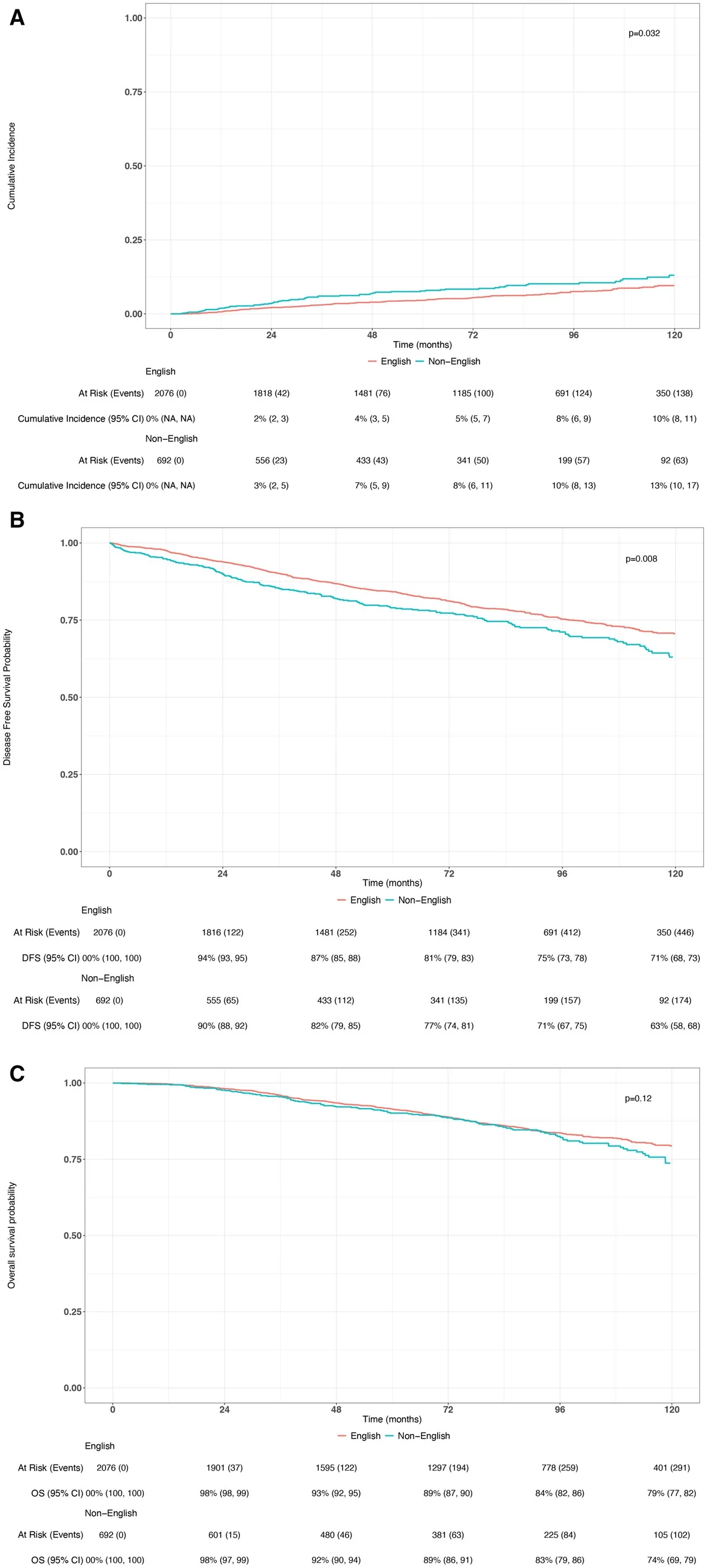
Among propensity-matched English and non-English speakers, LRR risk (A), DFS (B), and OS (C).

The cumulative incidence of LRR at 10 years was 9.5% among ES patients (95% CI = 8.0% to 11%) vs 13% in NES patients (95% confidence interval [CI] = 9.9% to 17%) (hazard ratio [HR] = 1.38, 95% CI = 1.03 to 1.84, *P* = .028). At 10 years, DFS was 71% (95% CI = 68% to 73%) in the ES group vs 63% (95% CI = 58% to 68%) in the NES group (HR = 1.26, 95% CI = 1.06 to 1.50, *P* = .008), whereas OS was 79% in the ES group (95% CI = 77% to 82%) vs 74% in the NES group (95% CI = 69% to 79%) (HR = 1.19, 95% CI = 0.95 to 1.48, *P* = .12) (Figure 1). In a sensitivity analysis further adjusting the models for race, ethnicity, marital status, religion, and insurance status, findings were directionally consistent with the primary analysis, LRR (HR = 1.32, 95% CI = 0.96 to 1.82), DFS (HR = 1.08, 95% CI = 0.88 to 1.33), and OS (HR = 1.07, 95% CI = 0.83 to 1.40).

The median time to death or last follow-up was 85.4 months for ES patients (range = 0.5-360.7) vs 77.1 months in NES patients (range = 1.5-357.8), whereas the median time to recurrence of any type, death, or last follow-up was 80.8 (range = 0.1-350.5) months vs 70.7 (range = 0.2-318.3) months.

## Discussion

In this large matched cohort study, we found that NES patients diagnosed with stage I-III breast cancer experienced significantly higher rates of LRR and lower DFS compared with matched ES counterparts, despite receiving curative care at a single, well-resourced NCI-designated Comprehensive Cancer Center with robust institutional language-access services, including professional on-site, telephone, and video interpreter support. The inferior DFS for NES patients did not translate into a statistically significant difference in OS, which may reflect the long natural history of stage I-III breast cancer and the potential effectiveness of salvage therapies following recurrence.

Studies have documented that patients with LEP face barriers across the cancer care continuum, and these barriers likely reflect broader structural inequities. Prior literature suggests that language-related disparities may arise through multiple pathways, including reduced access to screening and specialty care, delays in diagnosis and treatment, lower engagement with patient portals and clinical research, underuse or inconsistent use of professional interpretation, and difficulty navigating complex treatment recommendations in the setting of co-existing socioeconomic and immigration-related barriers.^1-4^ Even in settings where interpreter services are available, inequities may persist because language barriers often coexist with differences in health literacy, insurance, transportation, employment constraints, and other social needs that affect care continuity and treatment adherence.^5-8^ Accordingly, the persistent differences in LRR and DFS observed after matching should not be interpreted as showing that language proficiency itself is the sole driver of inequity. Rather, preferred language may serve as a marker of intersecting communication, access, and social barriers that remain incompletely addressed even within a well-resourced academic center, including lower rates of electronic patient portal enrollment,^9^ less clinical trial engagement,^9^ underuse of professional interpretation,^10^ cultural discordance,^10^ and broader structural barriers outside the clinic.^10^

Previous studies have focused primarily on access to care and initial treatment disparities, whereas our study is the first to directly compare long-term oncologic outcomes between ES and NES patients with breast cancer. Notably, disparities in health outcomes persisted even after matching for key clinical characteristics, suggesting that language proficiency could reflect broader challenges that contribute to differences in oncologic outcomes.

These findings have broad implications in an increasingly multilingual population.^11^ Even in highly resourced academic centers with comprehensive language access services, our study demonstrated that differences in cancer outcomes can persist, which highlights the need for continued attention to the nuances of communication, cultural challenges, and care delivery in populations that include LEP patients.

### Strengths and limitations

Our study includes a large propensity-matched cohort with long follow-up and clinically meaningful oncologic endpoints. The single-center setting reduced heterogeneity in treatment environment and institutional language-access resources, and this analysis is among the first to directly evaluate long-term oncologic outcomes rather than focusing on screening, access, or treatment delays.

This analysis must be interpreted in the context of the study design. As noted, this retrospective, single-center design limits causal inference and generalizability. Although patients were matched on key clinicopathologic factors, residual confounding remains likely because preferred language co-occurs with social determinants of health (including socioeconomic status, insurance, transportation, immigration-related factors, and health literacy) not comprehensively captured in the electronic medical record. The exposure reflected patient-reported preferred language rather than formal proficiency, precluding assessment of bilingual fluency or acculturation. Encounter-level interpreter utilization was not consistently captured and could not be evaluated as a modifier of outcomes. Finally, language-specific outcome analyses were not feasible given limited event counts within individual language subgroups.

## Conclusions

Despite matching for key clinicopathologic and treatment factors, we observed differences in oncologic outcomes between ES and NES patients with breast cancer, suggesting that language could serve as a proxy for broader structural and social barriers to health-care access and treatment adherence.^12^^,^^13^ Differences in health outcomes persisted even within a single academic cancer center with robust institutional resources, including readily available interpreter services and comprehensive multilingual patient support infrastructure.^14^ Importantly, our findings suggest that language proficiency may not be an isolated variable but rather one that potentially accompanies other social determinants of health to influence treatment outcomes.^15^ Addressing these adverse outcomes will require additional study and targeted interventions to recognize the nuanced survivorship challenges faced by NES patients.

## Data Availability

Data cannot be shared for ethical/privacy reasons.
